# Molecular Details of Retinal Guanylyl Cyclase 1/GCAP-2 Interaction

**DOI:** 10.3389/fnmol.2018.00330

**Published:** 2018-09-19

**Authors:** Anne Rehkamp, Dirk Tänzler, Claudio Iacobucci, Ralph P. Golbik, Christian H. Ihling, Andrea Sinz

**Affiliations:** ^1^Department of Pharmaceutical Chemistry and Bioanalytics, Charles Tanford Protein Center, Institute of Pharmacy, Martin Luther University Halle-Wittenberg, Halle, Germany; ^2^Department of Microbial Biotechnology, Charles Tanford Protein Center, Institute of Biochemistry and Biotechnology, Martin Luther University Halle-Wittenberg, Halle, Germany

**Keywords:** cross-linking, guanylyl cyclase-activating protein (GCAP), interaction site, mass spectrometry, photo-transduction, rod outer segment guanylyl cyclase (ROS-GC1)

## Abstract

The rod outer segment guanylyl cyclase 1 (ROS-GC1) is an essential component of photo-transduction in the retina. In the light-induced signal cascade, membrane-bound ROS-GC1 restores cGMP levels in the dark in a calcium-dependent manner. With decreasing calcium concentration in the intracellular compartment, ROS-GC1 is activated via the intracellular site by guanylyl cyclase-activating proteins (GCAP-1/-2). Presently, the exact activation mechanism is elusive. To obtain structural insights into the ROS-GC1 regulation by GCAP-2, chemical cross-linking/mass spectrometry studies using GCAP-2 and three ROS-GC1 peptides were performed in the presence and absence of calcium. The majority of cross-links were identified with the *C*-terminal lobe of GCAP-2 and a peptide comprising parts of ROS-GC1's catalytic domain and *C*-terminal extension. Consistently with the cross-linking results, surface plasmon resonance and fluorescence measurements confirmed specific binding of this ROS-GC peptide to GCAP-2 with a dissociation constant in the low micromolar range. These results imply that a region of the catalytic domain of ROS-GC1 can participate in the interaction with GCAP-2. Additional binding surfaces upstream of the catalytic domain, in particular the juxtamembrane domain, can currently not be excluded.

## Introduction

The retinal guanylyl cyclase 1 (ROS-GC1) is a transmembrane protein enabling the light adaption process in the eye's rods and cones (Koch, 1991; Dizhoor et al., 1994; Goraczniak et al., 1994). Dimeric ROS-GC catalyzes the conversion of GTP into cGMP in order to restore cGMP levels in the dark (Koch and Stryer, 1988). After light excitation of rhodopsin, a signal cascade induces the decrease of the intracellular cGMP concentration, resulting in the reduction of the intracellular calcium concentration to ~50 nM (Gray-Keller and Detwiler, 1994). Low calcium concentrations result in the activation of ROS-GC1 by the guanylyl cyclase-activating proteins 1/2 (GCAP-1/-2) that bind to the 68-kDa intracellular ROS-GC1 domain (Koch et al., 2002). This process is known as phototransduction (Schwartz, 1985; Pugh and Cobbs, 1986). The review by Schwartz (1985) addresses biophysical and electrophysiological properties of the, at that time so-called, “light-sensitive current.”

The GCAPs proteins (GCAP-1 and GCAP-2) belong to the neuronal calcium sensor (NCS) proteins that have four characteristic EF-hands. Three of them bind calcium, depending on the intracellular calcium concentration (Palczewski et al., 1994; Dizhoor et al., 1995; Burgoyne and Weiss, 2001). GCAP proteins are active in their calcium-free states and exist as *N*-terminally myristoylated forms (Hwang and Koch, 2002a). For GCAP-1, the *N*-terminal myristoyl group affects the protein's calcium sensitivity and activity, although a typical calcium-myristoyl switch, as observed for recoverin, has not been reported for GCAPs (Otto-Bruc et al., 1997; Hwang and Koch, 2002b). In case of GCAP-2, it has been suggested that myristoylation has little influence on the calcium-dependent activation of ROS-GC (Olshevskaya et al., 1997; Hwang and Koch, 2002b). Structures of myristoylated GCAP-1 (Stephen et al., 2007) and non myristoylated GCAP-2 (Ames et al., 1999) have been solved, however to date, no high-resolution structure is available for ROS-GC1.

The exact mechanisms of how GCAP-1 and GCAP-2 activate their target proteins are currently not understood. In particular, the surfaces within ROS-GC1 that interact with GCAP-1/-2 are controversially discussed (Laura and Hurley, 1998; Lange et al., 1999; Sokal et al., 1999; Duda et al., 2005; Peshenko et al., 2015a,b). Several studies indicate that both GCAP proteins share the same interaction sites in the kinase homology domain of ROS-GC1 and implicate that GCAP-1 and GCAP-2 compete for binding (Laura and Hurley, 1998; Peshenko et al., 2015b). In addition, Peshenko et al. showed that the ROS-GC dimerization domain participates in GCAP binding and thus the regulation of the human ROS-GC1 (Peshenko et al., 2015a). Another report suggests that the dimerization domain is not of vital importance for the activator binding, but may be involved in the calcium-dependent signal transduction (Zägel et al., 2013). An alternative point of view is that the juxtamembrane and kinase homology domain (KHD) of ROS-GC's intracellular domain represent the binding site of GCAP-1, while GCAP-2 interacts at the *C*-terminal region of the catalytic domain (Lange et al., 1999; Duda et al., 2005).

Moreover, it has been shown that dimerization of the catalytic domain can occur in the absence of the signal helix domain (SHD, also termed dimerization domain) as GCAP-2 can activate a ROS-GC1 mutant lacking the SHD (Duda et al., 2012). The ^657^WTAPELL^663^ motif in the *C*-terminal part of the KHD of ROS-GC1 engages in signal transfer processes to activate the catalytic domain, but not in the binding reaction to GCAPs (Duda et al., 2011). The hypothesis of separate interaction sites for GCAP-1 and GCAP-2 at ROS-GC1 is supported by affinity determinations via backscattering interferometry using different constructs of the intracellular domain (Sulmann et al., 2017).

To characterize protein interactions, complementary strategies, such as the cross-linking/mass spectrometry (MS) approach can be of advantage (Sinz, 2006, 2018; Rappsilber, 2011; Leitner et al., 2016). Cross-linking reagents covalently connect functional groups of amino acids located at a specific distance that can be bridged by the cross-linker. The identification of cross-linked products can be performed in a “bottom-up” approach, in which the cross-linked sample is enzymatically digested and subsequently analyzed by liquid chromatography coupled with tandem mass spectrometry (LC/MS/MS). Previously, cross-linking/MS studies using GCAP-2 and a ROS-GC peptide, derived from the *C*-terminal extension of the catalytic domain, allowed to define the structure of a GCAP-2/ROS-GC peptide complex in its Ca^2+^-bound state (Pettelkau et al., 2012).

**Figure 1:**
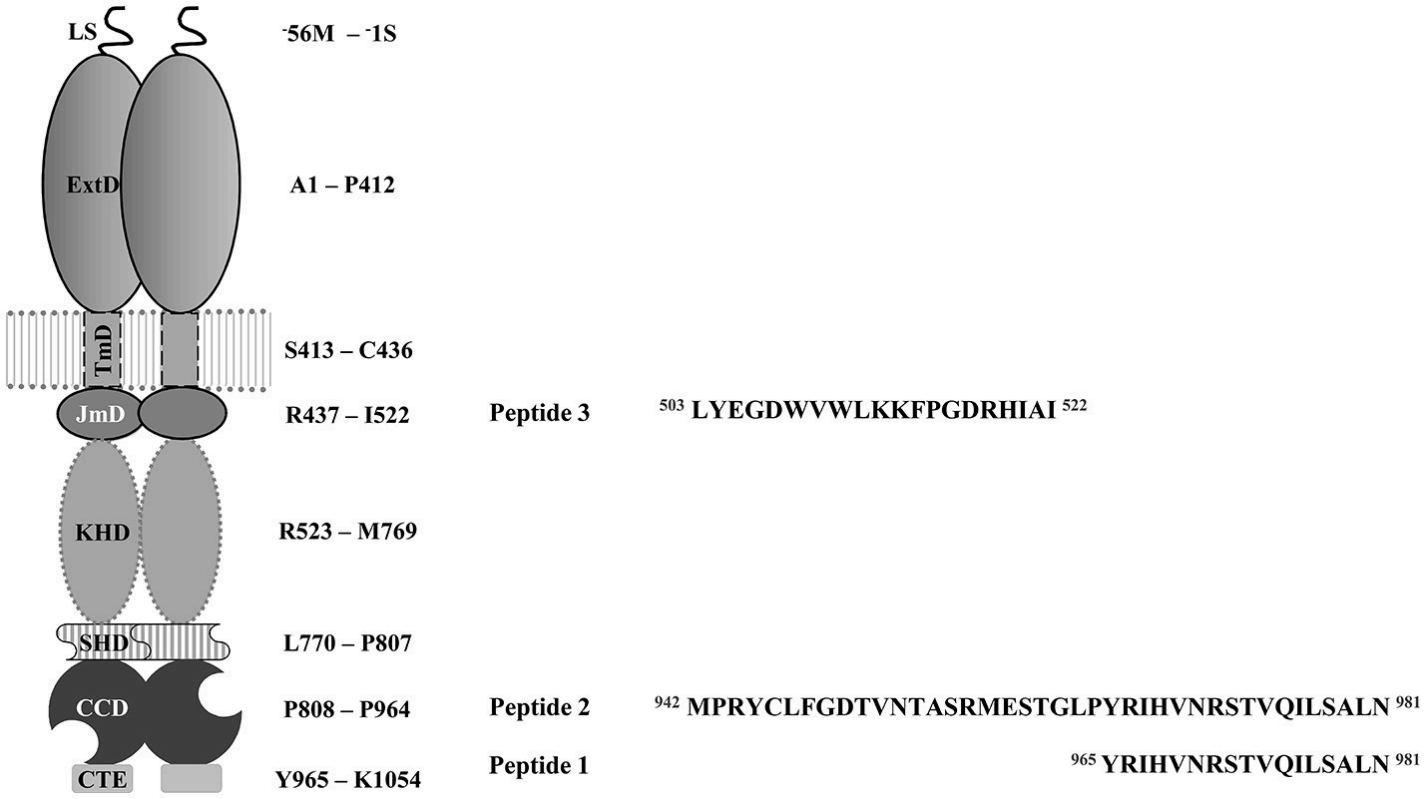
Schematic overview on the modular structure of the rod outer segment guanylyl cyclase 1 (ROS-GC1) dimer. Domain nomenclature is adapted from Duda et al. (2016). LS stands for leader sequence, ExtD, extracellular domain; TmD, transmembrane domain; JmD, juxtamembrane domain; KHD, kinase homology domain; SHD, signaling helix domain; CCD, core catalytic domain and CTE, *C*-terminal extension. Peptides are derived from the intracellular domain of ROS-GC. Peptide 1 is the postulated binding motif of GCAP-2 (aa 965-981) used in previous studies (Pettelkau et al., 2012). Peptide 2 (aa 942-981) is an *N*-terminal extension of peptide 1 comprising a part of the catalytic domain. Peptide 3 (aa 503-522) is the potential binding motif of GCAP-1 (Lange et al., 1999). Amino acid sequences of all three peptides are displayed.

To gain further insights into ROS-GC1 activation via GCAP-2, we extended these initial cross-linking studies with the aim to clarify whether GCAP-1 and -2 possess overlapping or separate binding sites in the intracellular region of ROS-GC1. To this end, three ROS-GC peptides were employed (Figure 1): Peptide 1 comprises the GCAP-2 binding motif (aa 965-981) (Duda et al., 2005), peptide 2 resembles peptide 1 with an *N*-terminal extension (aa 942-981), and peptide 3 represents the putative binding motif of GCAP-1 (aa 503-522) (Lange et al., 1999). For our cross-linking studies, we used the *in-house* developed MS/MS cleavable urea-based cross-linker disuccinimidyl dibutylurea (DSBU) (Müller et al., 2010) as well as the “zero-length” cross-linker *1, 1*′-carbonyldiimidazole (CDI) (Hage et al., 2017). The spacer arms of DSBU and CDI are 12.5 and 2.6 Å, respectively. DSBU mainly reacts with amine groups of lysines, while CDI reacts with both amine groups as well as hydroxyl groups of serines, threonines and tyrosines. The 1,3-diallylurea (DAU) cross-linker with a spacer length of ~10 Å connects exclusively cysteines (Iacobucci et al., 2018b). In addition, the artificial, diazirine-containing amino acid photo-methionine was incorporated into GCAP-2 to gain complementary structural information on the ROS-GC1 interaction by UV-induced cross-linking (Suchanek et al., 2005; Piotrowski et al., 2015). In order to quantify the interaction between GCAP-2 and ROS-GC1 peptides surface plasmon resonance (SPR) and fluorescence measurements were employed.

In this work, we provide insight into the GCAP-2 binding sites within ROS-GC1 by chemical cross-linking/MS. The majority of cross-links with ROS-GC1 peptide 2 were obtained with the C-terminal lobe of GCAP-2. Via SPR and fluorescence measurements, a dissociation constant in the micromolar range was determined.

## Materials and methods

### Expression and purification of GCAP-2

For the expression and purification of bovine GCAP-2, an existing protocol was modified (Schröder et al., 2011). GCAP-2 was expressed in *E*. *coli* BL21 (DE3) cells from vector pET-11a. For *in vivo* myristoylation, plasmid pBB131 encoding the yeast *N*-myristoyltransferase I was co-expressed. 60 μg/ml myristate (Thermo Fisher Scientific) was added to the culture at an OD_600_ of 0.4 (Duronio et al., 1989). At OD_600_ = 0.6 and 37°C, recombinant gene expression was induced with 1 mM IPTG for 4 h. For photo-methionine (photo-Met, Thermo Fisher Scientific) labeling, the compound was added (30 mg/l) at the time of induction. Further incubation was performed in the dark. The procedure of photo-Met incorporation was performed with slight adaptations according to an established protocol (Piotrowski et al., 2015). After harvesting the cells, inclusion bodies were isolated and solubilized. Briefly, the cell pellet was dissolved in 0.1 M TRIS-HCl and 1 mM EDTA (pH 7.0) and cell disruption was performed via French press. The disrupted cells were diluted in half the volume of buffer, consisting of 60 mM EDTA, 6% triton X-100, 1.5 M NaCl (pH 7.0), and incubated on ice for 30 min. Inclusion bodies were centrifuged, the pellet was washed four times with 0.1 M TRIS-HCl and 20 mM EDTA (pH 8.0) and solubilized in 6 M urea. Protein refolding was performed by dialysis against 50 mM TRIS-HCl, 1 mM CaCl_2_, and 1 mM TCEP (pH 8.0). GCAP-2 was purified by anion-exchange chromatography (HiTrap Q Sepharose HP, GE Healthcare), via a gradient from 0 to 1 M NaCl in 50 mM TRIS-HCl, 1 mM TCEP (pH 8.0), followed by size exclusion chromatography (Superdex 75 pg, 16/600, GE Healthcare) applying 10 mM HEPES, 150 mM NaCl, 2.5 mM TCEP, 10% glycerol (pH 7.5). To separate non-myristoylated from myristoylated GCAP-2, a reverse phase Agilent Eclipse XDB-C8 column (4.6 × 150 mm, 5 μm, and 1 ml/min) was used. Recombinant GCAP-2 was eluted by a gradient from 0 to 100% acetonitrile in 0.1% TFA. After drying and resuspensing in 6 M urea, the refolding process was repeated via dialysis in 20 mM HEPES, 1 mM TCEP (pH 7.5) (Hwang and Koch, 2002a). The purity of the protein was confirmed by ESI-MS.

### Cross-linking experiments

For all experiments, the myristoylated form of GCAP-2 and peptides (Figure 1) derived from bovine ROS-GC1 were used. For the cross-linking reactions with DSBU, CDI (Carbolution Chemicals), GCAP-2 and ROS-GC peptides were used at final concentrations of 10 μM in 20 mM HEPES buffer (pH 7.5). First, GCAP-2 was incubated at room temperature for 10 min in the presence of 1 mM CaCl_2_ or 10 mM EGTA to obtain the calcium-loaded, non-activating or the calcium-free, activating forms. Before the cross-linking reactions were initiated by adding the cross-linker (stock solution freshly prepared in DMSO, DSBU with a 100-fold and CDI with a 20-fold molar excess over GCAP-2), the samples were incubated with ROS-GC peptides (Thermo Fisher Scientific) for further 30 min. After 30 min at room temperature, the cross-linking reactions were stopped by addition of 20 mM ammonium bicarbonate (DSBU samples) and 0.5 M TRIS/HCl, (pH 8.0) (CDI samples). For cross-linking with DAU, GCAP-2 was incubated with the ROS-GC peptide 2 as described for cross-linking with DSBU and CDI. A 100-fold excess of DAU (Iacobucci et al., 2018b) and a 20-fold excess of the photo-radical inducer benzophenone (freshly prepared in DMSO) were added. The cross-linking reaction was performed by UV-A exposure (8,000 mJ/cm^2^) on ice and quenched with 5 mM dithiothreitol (DTT). For photo-Met cross-linking, labeled GCAP-2 was used at a concentration of 10 μM in 20 mM HEPES-buffer (pH 7.5). After 10 min of incubating GCAP-2 at room temperature in the presence of CaCl_2_ (final concentration 1 mM) or EGTA (final concentration 10 mM), ROS-GC peptides were added at a 10-fold molar excess (final concentration 100 μM) followed by further 30 min of incubation. The cross-linking reaction on ice was induced by irradiation with UV-A light (365 nm, 8,000 mJ/cm^2^). In case the samples were not immediately applied, they were stored at −20°C.

### SDS-PAGE analysis and proteolysis

To separate cross-linked species from non-reacted proteins, 4–20% gradient gels (Mini-PROTEAN TGX Gel, Biorad) were applied. The DSBU cross-linked and predicted GCAP-2-peptide (1:1) complexes were excised from the gel, reduced with dithiothreitol (DTT) and carbamidomethylated with iodacetamide (IAA). For *in-solution* digestion, after denaturation by sodium deoxycholate, CDI and photo-Met cross-linked samples were prepared according to an existing protocol with slight adaptations (Lössl and Sinz, 2016). DSBU-, CDI-, or DAU- cross-linked samples, were incubated overnight with GluC (1:20, enzyme:protein ratio) at 37°C. Photo-Met-cross-linked samples were digested with AspN (1:50 ratio). Subsequently, the samples were digested for 4 h with 250 ng trypsin (all proteases from Promega).

### LC/MS/MS

Separation of the proteolytic peptide mixtures was performed via the Ultimate 3000 RSLC Nano system (Thermo Fisher Scientific). As precolumn, a C8 reversed phase (RP) (Acclaim PepMap, 300 μM ^*^ 5 mm, 5 μm, 100 Å, Thermo Fisher Scientific) was employed. As separation columns, C18 RP (Acclaim PepMap, 75 μm ^*^ 250 mm, 2 μm, 100 Å, Thermo Fisher Scientific) or PicoFrit C18 nanospray columns (75 μm ID, 10 μm tip, New Objective, packed with ReproSil-Pur 120 C18-AQ, 1.9 μm) were employed. The nano-HPLC system was coupled to the nano-ESI source of the Orbitrap Fusion Tribrid or the Orbitrap Q-Exactive Plus mass spectrometer (Thermo Fisher Scientific). The samples were desalted on the pre-column by 0.1% trifluoroacetic acid (TFA) for 15 min. Solvent A was H_2_O (LC-MS grade, VWR) with 0.1% formic acid (FA), Solvent B consisted of 80% acetonitrile (LC-MS grade, VWR) and 0.08% FA. For peptide separation, an elution gradient (flow rate 300 nl/min) was set to 35% solvent B in 90 min, according to a previous study (Iacobucci et al., 2018b). The data-dependent MS/MS mode was applied for data acquisition. For MS/MS, the most intense signals within 5 s of the previous full MS scan were isolated (isolation window 2 u) and fragmented by higher-energy collision-induced dissociation (HCD) (normalized collision energy 30 ± 3%). The fragment ions were analyzed in the orbitrap. Xcalibur 4.0.27 (Thermo Fisher Scientific) was used to control the data acquisition.

### Identification of cross-linked products

Cross-linked products were identified with the *in*-*house* developed software StavroX (version 3.6.0.1) and MeroX (version 1.6.0.1) (Götze et al., 2012, 2015). The software employs Mascot generic format (mgf) files for data analysis. The maximum mass deviations for precursor and fragment ions were fixed at 3 and 10 ppm. The signal-to-noise ratio was ≥2.0. The following settings were applied to define enzymatic cleavage sites: *C*-terminal to K and R (for trypsin), *C*-terminal to D and E (for GluC), *N*-terminal to D and E (for AspN). Three missing cleavage sites were allowed for amino acid residues K, R, D, and E. For the DSBU and CDI cross-linkers, one cross-linking site was defined for K, the second one for K, S, T, Y and *N*-termini. Photo-Met can react with all amino acids, while DAU only connects C. All cross-links suggested by the software were manually validated. 3D-protein structures were visualized by PyMOL (0.99rc6, Schrödinger LLC) and cross-links were presented as circular plots by Circos (0.67-7) (Krzywinski et al., 2009).

### Surface plasmon resonance (SPR) measurements

For SPR measurements, the MP-SPR Navi 200 OTSO system (BioNavis) was employed in order to determine dissociation constants for the GCAP-2/ROS-GC peptide interactions. For all measurements 2D (planar) carboxymethyldextran (CMD) hydrogel-coated sensor slides (SPR102-CMD-2D, BioNavis) were applied. All buffers were degassed. The running buffer for the immobilization and peptide measurements consisted of 20 mM HEPES, 0.05% Tween (pH 7.5). The regeneration step occurred via addition of 10 mM glycine, 0.05% Tween (pH 2.0). The flow rate was set to 30 μl/min and the temperature was set to 22°C. GCAP-2 was immobilized by amine coupling. To this end, the chip surface was activated by the 0.4 M EDC/0.1 M NHS (freshly prepared). Activation was repeated twice with 250 μl solution. 5 μM GCAP-2, which had been diluted in 10 mM sodiumacetate buffer (pH 3.9), was injected twice for immobilization on the CMD sensor surface. Non-reacted NHS ester groups were deactivated with 1 M ethanolamine injection. For binding measurements, ROS-GC peptides [final concentrations 0.5, 1, 5, 10, 20, and 40 μM (peptides 1 and 2); 5, 10, 20, and 40 μM (for peptide 3)] were diluted in running buffer. The injection time was 420 s at a flow rate of 30 μl/min. SPR Navi control and SPR Navi Data Viewer (BioNavis) were used to control the SPR measurements. Data analysis was performed via the kinetic evaluation tool by the software TraceDrawer (version: 1.8, Ridgeview Instruments AB). The graphs presented in Figure 4 were generated with OriginPro 2018 (Northampton, MA). To describe the binding behavior between GCAP-2 and ROS-GC peptides 1 and 2, a two-state model with equimolar binding was applied. This interaction model represents an initial interaction event, which then changes into an alternative interaction. While the initial binding is assumed to be weak, the primary complex will rearrange into a secondary complex with stronger interaction.

(1) $$\frac{\text{d}\left[\text{B}\right]}{\text{dt}}=\;k_{\text{d1}}\cdot \left[\text{AB}\right]\text{ − }k_{\text{a1}}\cdot \left[\text{A}\right]\cdot \left[\text{B}\right]$$

(2) $$\frac{\text{d}\left[\text{AB}\right]}{\text{dt}}=\;k_{\text{a1}}\cdot \left[\text{A}\right]\cdot \left[\text{B}\right]\text{ - }k_{\text{d1}}\cdot \left[\text{AB}\right]\text{ - }k_{\text{a2}}\cdot \left[\text{AB}\right]\text{ + }k_{\text{d2}}\cdot \left[\text{A}{\text{B}}^{\prime }\right]$$

(3) $$\frac{\text{d}\left[\text{A}{\text{B}}^{\prime }\right]}{\text{dt}}=\;k_{\text{a2}}\cdot \left[\text{AB}\right]\text{ - }k_{\text{d1}}\cdot \left[\text{A}{\text{B}}^{\prime }\right]$$

(4) $$\text{Y }\sim \;\left[\text{AB}\right]\text{ + }\left[\text{A}{\text{B}}^{\prime }\right]$$

The signal amplitude is proportional to the sum of the concentrations of the two complexes AB and AB′.

(5) $$\text{At t = 0 }\to \;\left[\text{AB}\right]\;=\left[\text{A}{\text{B}}^{\prime }\right]\text{ = 0 and }\left[\text{B}\right]\text{ = }{\left[\text{B}\right]}_{\max}$$

Y recorded signal
  Y_max_ maximum of the recorded signal
  [AB] concentration of the primary complex
  [AB′] concentration of the secondary complex
  [B] concentration of the unbound target
  [A] concentration of the ligand
  *k*_a1_ bimolecular association rate constant for primary complex formation
  *k*_a2_ monomolecular rate constant of the interconversion [*AB*] → [*AB*′]
  (secondary complex formation)
  *k*_d1_ monomolecular dissociation rate constant
  *k*_d2_ monomolecular rate constant of the interconversion [*AB*′] → [*AB*]

The equation is solved by numerical integration by the TraceDrawer software. Since the formation of the complex AB′ from AB does not cause any signal change, it is difficult to estimate the microscopic kinetic constants. The changes in the signal amplitude can be investigated according to the following equation to obtain the bimolecular association and the monomolecular dissociation rate constants.

(6) $$\frac{\text{dY}}{\text{dt}}=\;k_{\text{a1}}\cdot \left[\text{A}\right]\cdot {\text{Y}}_{\max}\text{ - }\left(k_{\text{a1}}\cdot \left[\text{A}\right]\text{ + }k_{\text{d1}}\right)\cdot Y\;=\;k_{a1}\cdot \left[A\right]\cdot \left(Y_{\max}\;-\;Y\right)\;-\;k_{d1\;}\cdot Y$$

(7) $$k_{\text{s}}\;=\;k_{\text{a1}}\cdot \left[\text{A}\right]\text{ + }k_{\text{d1}}$$

The plot of *d*Y/*d*t against Y will yield the term *k*_s_ as slope. The plot of *k*_s_ against [A] will yield a straight line with *k*_a1_ as slope and *k*_d1_ as intercept. Furthermore, the value *k*_d1_ can be determined from the dissociation curve according to the following equation:

(8) $$\ln\left(\frac{{\text{Y}}_{\text{0}}}{{\text{Y}}_{\text{t}}}\right)\;=\;k_{\text{d}}\cdot \left(\text{t }-{\text{t}}_{\text{o}}\right)$$

(9) $${\text{Y}}_{\text{t}}\;=\;{\text{Y}}_{\text{0}}\;\cdot \;\exp\left[-\;k_{\text{d}}\cdot \left(\text{t }-\;{\text{t}}_{\text{0}}\right)\right]$$

Y_0_ recorded signal at time t_0_

Y_t_ recorded signal at time t

The *K*_*D*_ value can be calculated according to

(10) $${\text{K}}_{\text{D }}=\;\frac{k_{\text{d}}}{k_{\text{a}}}$$

### Fluorescence measurements

Fluorescence measurements were carried out with a Jasco Spectrofluorometer FP-8200, equipped with a Jasco MCB-100 Mini Circulation Bath (20°C, xenon lamp, data interval 0.5 nm, accumulation number 3). The excitation wavelength was 280 nm, fluorescence emission was recorded at 350 nm to exclude self-fluorescence of non-bound peptide. For the measurements, GCAP-2 was mixed with increasing concentrations of ROS-GC peptides in 20 mM HEPES (pH 7.5). As a negative control, separate recordings were performed with *N*-acetyl-L-tryptophanamide or *N*-acetyl-L-tyrosinamide, respectively. The composition of the controls correlates with the peptides' composition of tryptophan and tyrosine residues to exclude that a change in fluorescence intensity results from self-fluorescence of the peptides.

## Results

To investigate the binding site of GCAP-2 at the intracellular domains of ROS-GC1, cross-linking reactions of myristoylated GCAP-2 and three ROS-GC1 peptides (Figure 1) were performed. Peptide 1 had been identified in previous cross-linking studies as a GCAP-2 binding segment, which formed the basis for deriving a model of the GCAP-2/peptide complex in the presence of calcium (Pettelkau et al., 2012). This peptide had been earlier suggested as a core binding site for GCAP-2 (Duda et al., 2005). In this work, we also studied an *N*-terminally extended version of peptide 1 (peptide 2) comprising a part of the catalytic domain in order to gain more detailed insights into the GCAP-2 binding site at ROS-GC1. In addition, peptide 3, representing a potential interaction site of GCAP-1 (Lange et al., 1999), was investigated to identify possible overlapping or additional binding sites of GCAP-1 and-2 in the juxtamembrane domain (Peshenko et al., 2015b).

### Chemical cross-linking strategies

Cross-linkers possessing diverse reactivities and spanning varying distances were used to covalently fix a GCAP-2/ROS-GC1 peptide complex in the presence (+Ca^2+^) and absence of calcium (−Ca^2+^). The DSBU cross-linker targets mainly primary amine groups, while CDI reacts with both amine and hydroxy groups. DAU reacts with sulfhydryl groups and incorporated photo-Met interacts with any amino acid, with a preference for the acidic amino acids, glutamic and aspartic acid (Iacobucci et al., 2018a). For DSBU cross-linking, the different cross-linked species were analyzed by SDS-PAGE (Supplementary Figure 1). In the presence of calcium, no clear signal was detected for the GCAP-2 control sample in the absence of cross-linker and peptide, which is caused by the presence of different calcium-loaded states. Consequently, an SDS-PAGE separation of GCAP/ peptide (1:1) complex and GCAP monomer bands was not completely possible, and for the in-gel digestion both signals (Ia/Ib) had to be excised *en bloc*. For CDI, DAU, and photo-Met, in-solution digestion was performed. It should be noted in this context that all four cross-linking reagents are MS/MS-cleavable and generate characteristic reporter ions to facilitate the identification of cross-linking reaction products. As such, the fragmentation patterns of the cross-linkers used herein prevent that isobaric species—originating from partially hydrolyzed cross-linker with consecutive peptide sequences—can be mistaken for “true” cross-links (Iacobucci and Sinz, 2017). For our studies, the automated cross-link identification performed by the MeroX software proved highly beneficial for a correct assignment of cross-linked products (Götze et al., 2015; Hage et al., 2017; Iacobucci et al., 2018a,b).

### Cross-linked products between GCAP-2 and ROS-GC1 peptides

**Figure 2:**
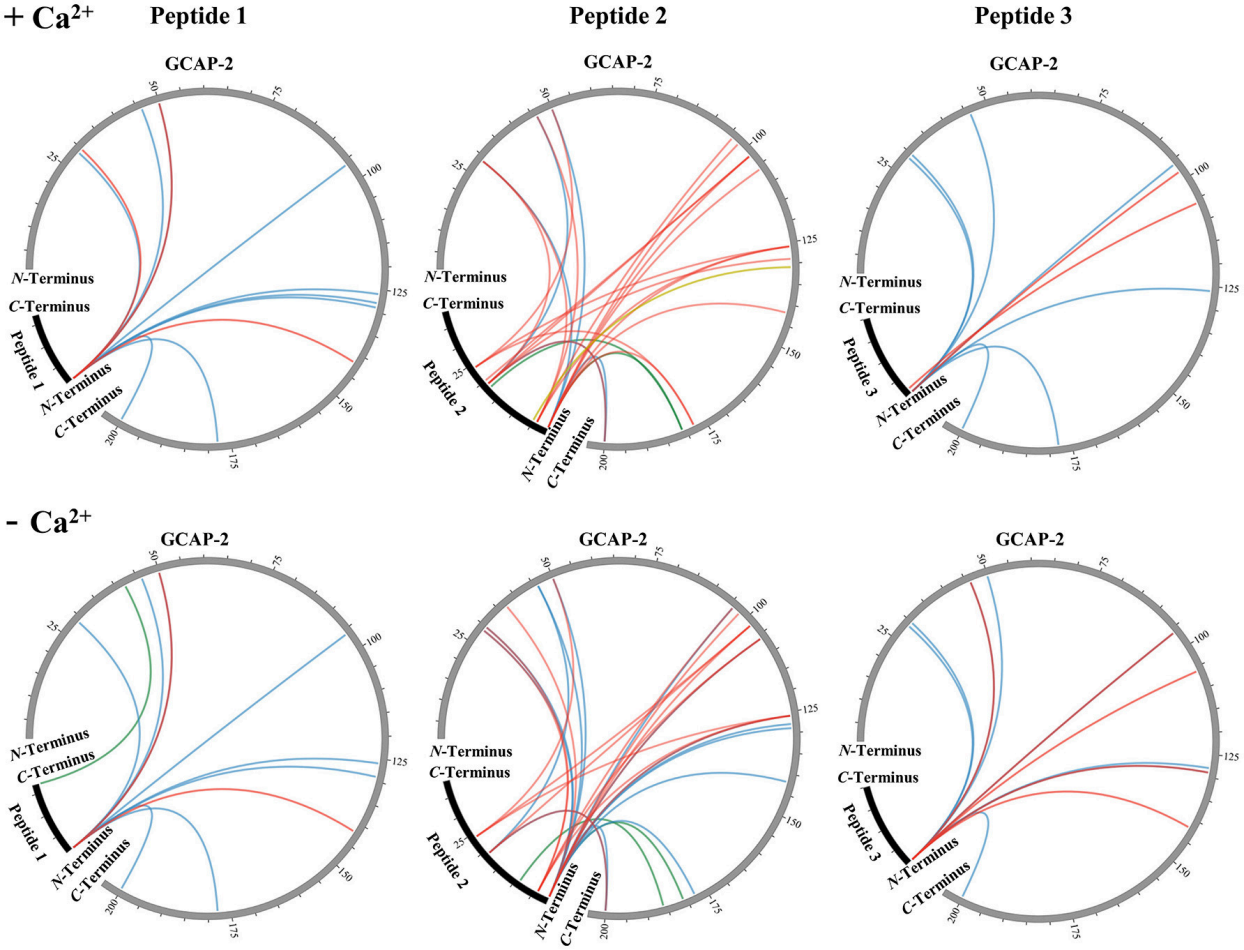
Unique cross-links between myristoylated GCAP-2 and ROS-GC peptides 1, 2, and 3 in the presence **(Upper)** and absence **(Lower)** of calcium (±Ca^2+^). DSBU cross-links are illustrated in blue, CDI cross-links in red, the DAU cross-link is labeled in yellow, and photo-Met cross-links are shown in green. The circular plots were generated by the Circos software (Krzywinski et al., 2009).

**Figure 3:**
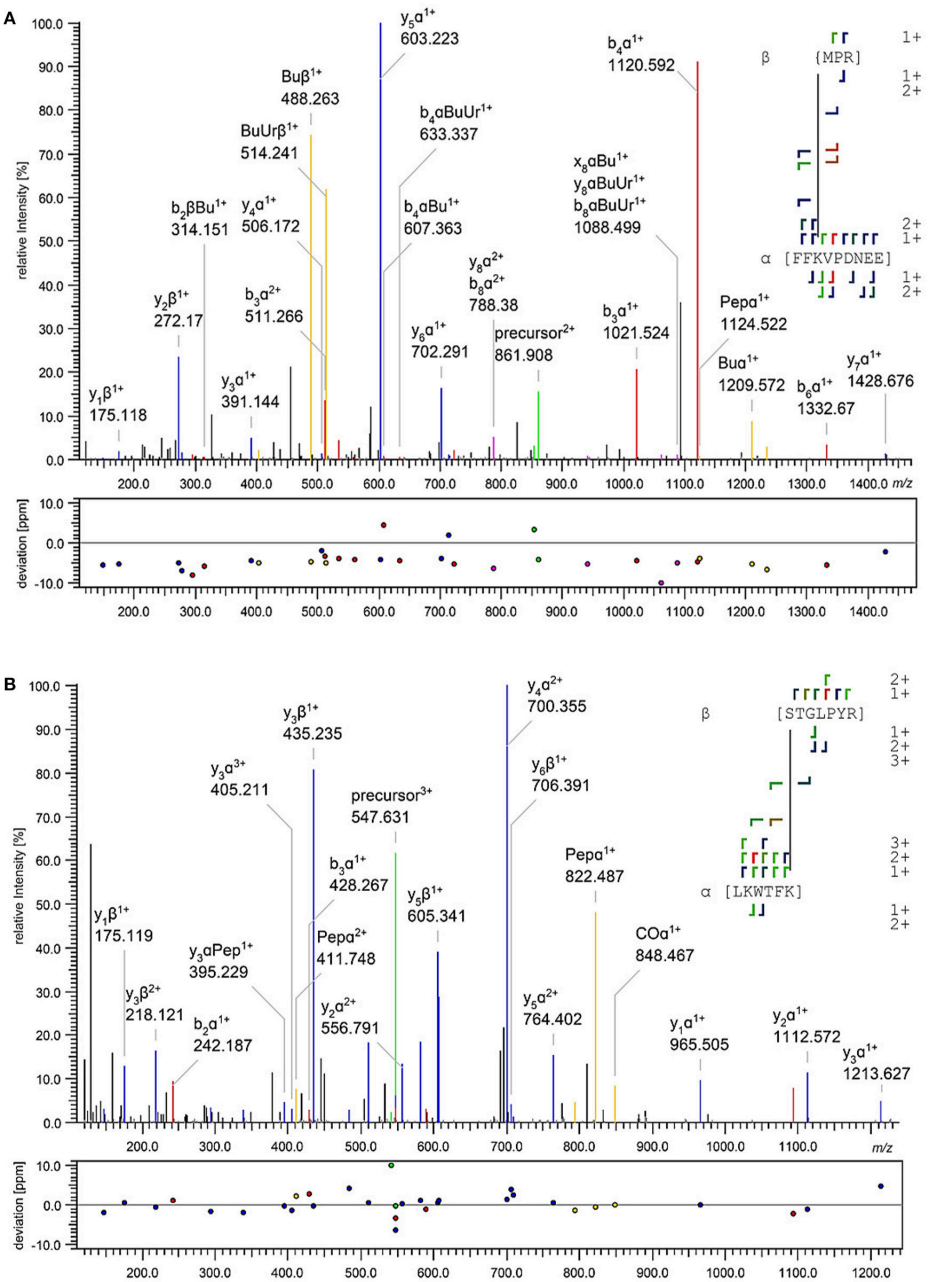
Exemplary fragment ion mass spectra of identified DSBU **(A)** and CDI **(B)** cross-links between GCAP-2 and peptide 2. The annotation was automatically performed by MeroX (Götze et al., 2015). A doubly charged precursor ion [M+2H]^2+^ at *m/z* 861.908 **(A)** and a triply charged precursor ion [M+3H]^3+^ at *m/z* 547.631 **(B)** were selected for HCD fragmentation. In **(A)**, the cross-link was identified between the *N*-terminus of peptide 2 (aa 1-3) and K50 of GCAP-2 (aa 48-56); in **(B)** the cross-link was identified between S19/T20 of peptide 2 (aa 19-25) and K102 of GCAP-2 (aa 97-102). Precursor ions are assigned in green, b- and y-type fragment ions in red and blue, and characteristic cross-linker fragment ions in yellow.

All unique cross-links identified between the GC-peptides and myristoylated GCAP-2 in its calcium-loaded, non-activating (+Ca^2+^, Figure 2, Upper) and calcium-free, activating state (–Ca^2+^, Figure 2, Lower) are visualized as circular plots to contrast calcium-dependent differences. The unique cross-linking sites are summarized in Table 1, all cross-links identified are listed in the Supplementary Tables 1–4. For peptide 1, the cross-linking sites between the *N*-terminus of the peptide and GCAP-2 are, with one exception, identical in the presence and absence of calcium. A single additional cross-link with photo-Met was identified in the absence of calcium. Peptide 2 has two main reactions sites, the first site comprising residues 1-9, the second one comprising residues 18-24. The latter site overlaps with the *N*-terminus of peptide 1 to confirm the cross-linking site of peptide 1. A DSBU cross-link between GCAP-2 and the *N*-terminus of peptide 2 and a CDI cross-link with S19/T20 are shown as representative examples of both reactions sites in Figures 3A,B. For peptide 2, the number of cross-links was similar in the non-activating (+Ca^2+^) and activating state (–Ca^2+^) of GCAP-2 (Table 1). DSBU cross-links (Figure 2, colored in blue) that can bridge up to 30 Å and thereby yield longer distances than CDI were identified at a higher frequency in the calcium-free than in the calcium-loaded state. These observations are in agreement with the previously published data using the BS^2^G cross-linker (Pettelkau et al., 2012). BS^2^G has comparable distance properties and can capture flexible structures, as does DSBU.

**Table 1:** Unique cross-linking sites between GCAP-2 and ROS-GC peptides 1-3 in the presence (1 mM CaCl_2_) and absence (10 mM EGTA) of calcium using DSBU, CDI, DAU, and photo-Met (PM) as cross-linkers.

**Site (1)**	**Site (2)**	**Calcium**	
**Peptide**	**mGCAP-2**	**With**	**Without**
**PEPTIDE 1**			
***C*-term**	**PM42**		✔
***N*-term**	**K126**	✔	✔
***N*-term**	**K128/129**	✔	✔
***N*-term**	**K178**	✔	✔
***N*-term**	**K200**	✔	✔
***N*-term**	**K29/30**	✔	✔
***N*-term**	**K46**	✔	✔
***N*-term**	**K50**	✔	✔
***N*-term**	**K96**	✔	✔
***N*-term**	**K142**	✔	✔
**PEPTIDE 2**			
**C5**	**C131**	✔	
**D9**	**PM186**	✔	✔
**E18**	**PM181**	✔	✔
**M1**	**PM181**	✔	✔
***N*-term**	**K102**	✔	✔
***N*-term**	**K106**	✔	✔
***N*-term**	**K128/129**		✔
***N*-term**	**K142**	✔	✔
***N*-term**	**K178**	✔	✔
***N*-term**	**K200**	✔	✔
***N*-term**	**K29/30**	✔	✔
***N*-term**	**K46**		✔
***N*-term**	**K50**	✔	✔
***N*-term**	**K96**		✔
***N*-term**	**K98**	✔	✔
***N*-term**	**S37**		✔
***N*-term**	**Y125/K126**	✔	✔
**S19**	**K200**	✔	✔
**S19**	**K46**	✔	✔
**S19/T20**	**K102**	✔	✔
**S19/T20**	**K129**	✔	
**S19/T20**	**K29**	✔	
**S19/T20/Y24**	**K200**	✔	✔
**Y24**	**K102**		✔
**Y24**	**K126**	✔	✔
**Y24**	**K178**	✔	
**Y24**	**K50**	✔	✔
**Y4**	**K106**		✔
**Y4**	**K126**	✔	✔
**Y4**	**K29/30**	✔	✔
**Y4**	**K46**	✔	
**Y4**	**K96/98**	✔	✔
**PEPTIDE 3**			
***N*-term**	**K126**	✔	
***N*-term**	**K128/129**		✔
***N*-term**	**K178**	✔	
*N*-term	K200	✔	✔
*N*-term	K29/30	✔	✔
*N*-term	K46	✔	✔
*N*-term	K50		✔
*N*-term	K96	✔	✔
*N*-term/Y2	K98	✔	
Y2	K106	✔	✔
Y2	K142		✔

In contrast, CDI (Figure 2, colored in red), an ultra-short cross-linker bridging distances up to maximally ~16 Å, was employed to obtain complementary structural information. Cross-links between lysines in the *C*-terminal part of GCAP-2 (K129-K200) and peptide 2 were mainly identified in the calcium-loaded state of GCAP-2. Calcium-independent CDI cross-links with peptide 2 were observed for all lysine residues in the amino acid sequence stretch K96-K126 of GCAP-2. We speculate that residues K96-K126 in GCAP-2 represent a major interaction site with ROS-GC. This is underlined by the fact that the cross-links with photo-Met and DAU point to the same region (Figure 5). The DAU cross-link (C131 in GCAP-2 – C5 in peptide 2) was only found in the presence of calcium.

The number of cross-links with peptide 3 was comparable in the presence and absence of calcium (Figure 2). Strikingly, DSBU and CDI reacted exclusively with the *N*-terminus and Y2 of peptide 3 although it is the only peptide of this study containing two lysine residues that should be preferentially targeted by amine-reactive cross-linkers. No photo-Met cross-links were observed between GCAP-2 and peptide 3.

### Affinities of GCAP-2 and ROS-GC1 peptides

**Scheme 1:**
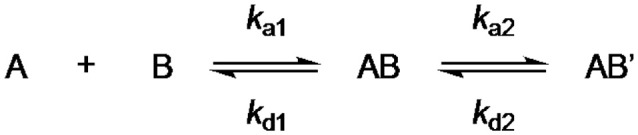
For the binding between GCAP-2 and ROS-GC peptides 1 and 2, a two-state model with equimolar binding was applied.

We performed surface plasmon resonance (SPR) measurements with GCAP-2 and the three ROS-GC1 peptides to determine K_D_ values of the protein/peptide interactions. GCAP-2 was immobilized on 2D-CMD chips, and peptides were injected at different concentrations. The kinetic evaluation was conducted according to a 1:1 binding model including two binding states and a conformational change upon binding (Scheme 1).

**Figure 4:**
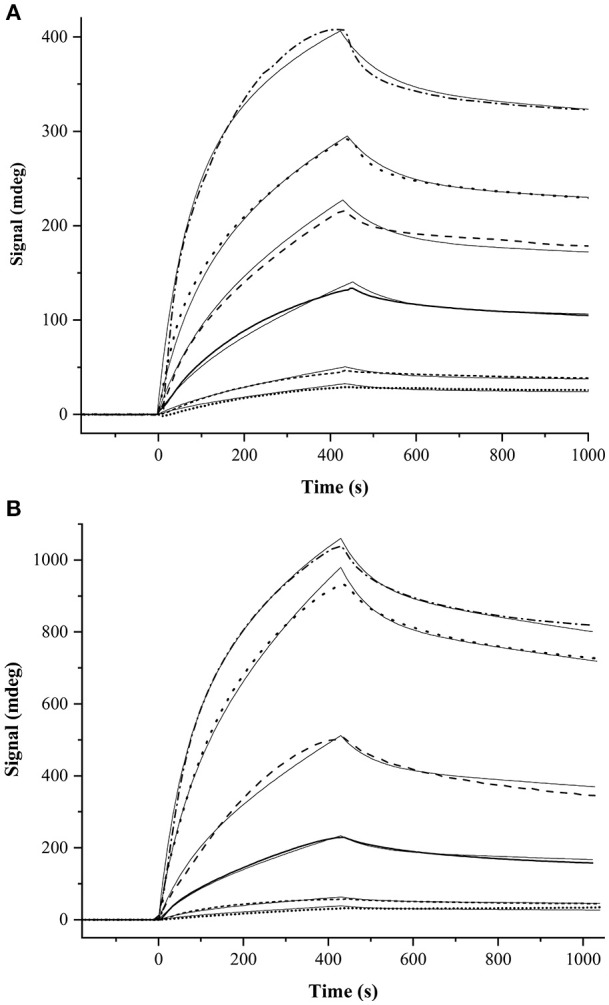
One set of SPR measurements between GCAP-2 and ROS-GC **(A)** peptide 1 and **(B)** peptide 2. The other SPR dataset is shown in (Supplementary Figure 3). The following peptide concentrations were applied: 0.5 μM (dotted line), 1 μM (dashed line), 5 μM (solid line), 10 μM (long-dashed line), 20 μM (long-dotted line), and 40 μM (dashed/dotted line). Curve fittings are shown as thin, solid lines. The K_D_ value of the interaction of GCAP-2 and peptide 1 is 1.12 ± 0.74 μM, while that of peptide 2 is 3.00 ± 0.59 μM.

For peptides 1 and 2, *K*_D_ values of 2.36 μM and 1.99 μM, respectively, were determined. These values are derived from two separate SPR measurements (Figure 4, Supplementary Figure 3). For peptide 3, the SPR curves could not be fitted suggesting that the interaction between GCAP-2 and peptide 3 is too weak to be detected with SPR.

Additionally, we conducted fluorescence spectroscopy by titrating peptides 1-3 to GCAP-2. A wavelength of 350 nm was chosen to rule out that changes in fluorescence originate from an increased concentration of non-bound peptide. Only peptides 1 and 2 delivered changes in fluorescence, while peptide 3 did not exhibit any changes compared to the negative control samples, *N*-acetyl-L-tryptophanamide or *N*-acetyl-L-tyrosinamide. We chose these compounds as controls as they resemble the aromatic composition of the ROS-GC1 peptide and as such, possess similar spectroscopic properties.

According to the fluorescence measurements, GCAP-2 binding only takes place for peptides 1 and 2. The K_D_ values between GCAP-2 and ROS-GC peptides 1 and 2 derived from fluorescence titration were in the same range as the values determined by SPR. For peptide 1, a K_D1_ value of 4.2 μM was observed (Supplementary Figure 2). The data were investigated by fitting according to an optimum behavior. Due to the susceptibility of the fitting routine in the first part, the value K_D1_ of peptide 2 was set to 1 μM by extrapolation of the starting point.

During titration of peptide 1 to GCAP-2, an initial increase and subsequent decrease in fluorescence were recorded, describing an optimal behavior. Both signal changes are related to peptide binding at different GCAP-2 sites, orginating from dequenching by the first binding event and quenching by a second event. The K_D_ values of the first and second binding events differ by two orders of magnitude with K_D2_ values of 244 μM (peptide 1) and 326 μM (peptide 2).

Finally, it has to be noted that for SPR and fluorescence measurements, calcium concentrations were not controlled by a chelator system. The calcium concentrations of the buffer solutions used for both measurements were determined by ICP-MS to be ~2 μM.

## Discussion

The calcium-dependent molecular regulation process of ROS-GC1 has so far remained elusive. In particular, the binding regions between ROS-GC1 and GCAP-2 are under debate: A mutational study predicted that ROC-GC1 activation by GCAP-2 requires the *N*-terminal part of the intracellular domain (residues M443-S746) of ROS-GC1 (Laura and Hurley, 1998). Binding of GCAP-2 upstream the catalytic domain was confirmed by *in vivo* studies (Peshenko et al., 2015b). These investigations involved ROS-GC1 mutants consisting solely of this region or with a deletion of a potential GCAP-2 binding motif in the *C*-terminal extension of the catalytic domain. This alternative binding region for GCAP-2 in the *C*-terminal extension of ROS-GC1 has been proposed upon activity and SPR measurements of further ROS-GC mutants (Duda et al., 2005). In addition, Sharma and co-workers observed that GCAP-2 could activate a ROS-GC mutant lacking the juxtamembrane and kinase homology domains (Duda et al., 2012).

In this work, the calcium-dependent binding of ROS-GC1 peptides to GCAP-2 was investigated by cross-linking/MS and biophysical methods. To resolve the debate of whether GCAP-2 binds to the membrane close or at the *C*-terminal part of ROS-GC1, three peptides were employed (Figure 1): Peptides 1 and 2 represent the *C*-terminal region beyond residue 942, while peptide 3 comprises residues 503-522 of the juxtamembrane part. Peptide 2 is an *N*-terminally extended version of peptide 1 that is thought to comprise two α-helices and two β-strands, according to homology models with a catalytic domain of rat type II adenylyl cyclase (PDB 1AWL) and green algae guanylyl cyclase (PDB 3ET6) (Liu et al., 1997; Winger et al., 2008). Peptide 2 comprises conserved motifs of the catalytic domain of mammalian guanylyl cyclases (Tucker et al., 1998; Winger et al., 2008; Ravichandran et al., 2017) and C946, N953, and R957 may be involved in GTP recognition and binding.

### Affinity between GCAP-2/ROS-GC1 peptides

In fact, affinities with K_D_ values in the low micromolar range were determined between GCAP-2 and peptides 1 and 2 via SPR and fluorescence measurements (Figure 4, Supplementary Figures 2, 3). In agreement with these findings, previous binding studies employing recombinant ROS-GC1 fragments spanning residues 733-1054 and 965-1054 had suggested K_D_ values of approximately 2 μM (Duda et al., 2005). For both SPR and fluorescence studies, 1:1 binding between GCAP-2 and ROS-GC1 peptides was assumed (Scheme 1). A 1: 1 binding model is consistent with MS analyses of GCAP-2 and peptide 1 (Pettelkau et al., 2012). Interestingly, under the settings used in the fluorescence measurements, second binding sites were recorded between GCAP-2 and each of the two ROS-GC1 peptides 1 and 2 with high K_D_ values (peptide 1: 244 μM and peptide 2: 326 μM), representing weak secondary interaction sites. We cannot completely rule out at the moment that these secondary binding events originate from possible ternary complexes between ROS-GC1 peptides and GCAP-2.

In comparison, K_D_ values determined for GCAP-2 and the human, full-length guanylyl cyclase (418 ± 135 nM) via backscattering interferometry, are consistent with the results shown herein (Sulmann et al., 2017). However, no interaction between GCAP-2 and the isolated domain of human ROS-GC-1 residues 496-806 (comprising the juxtamembrane and kinase homology domains) could be demonstrated in those experiments. This is consistent with our observation that no interaction was detected between peptide 3 (aa 503-522) and GCAP-2, neither in SPR nor in fluorescence experiments. Taken together, the affinities determined between GCAP-2 and peptides 1 and 2 are in a moderate range. Currently, additional binding surfaces that may arise from the flexible 3D-structure of the intracellular domain of ROS-GC1 cannot be excluded.

### Structural details of GCAP-2/ROS-GC1 peptide interaction

**Figure 5:**
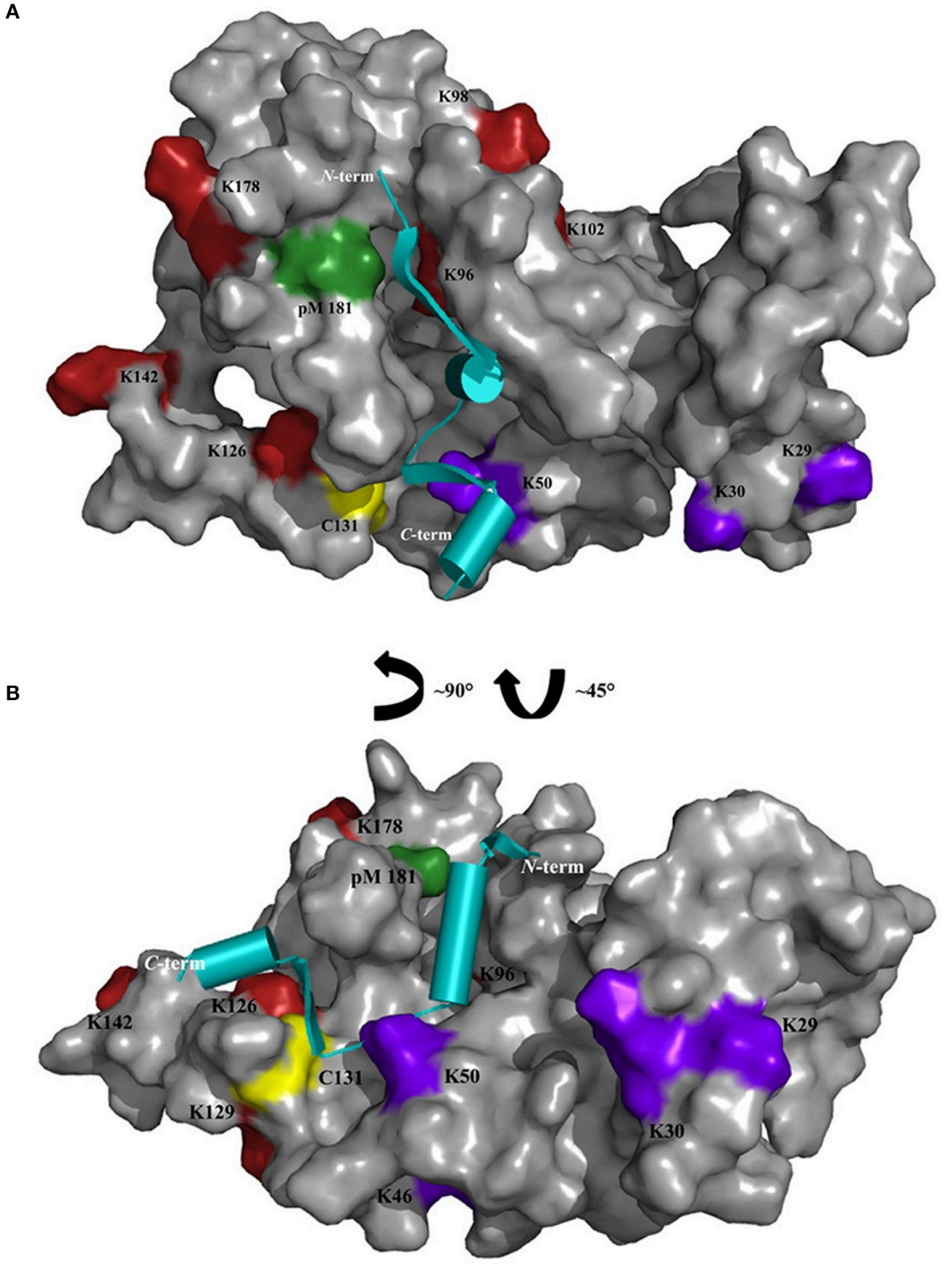
Cross-linked amino acids of GCAP-2 with peptide 2 are presented on the surface of non-myristoylated GCAP-2 (PDB 1JBA) in the presence of calcium; **(A)** front view, **(B)** side view. Cross-linked amino acids are colored as follows: DSBU and CDI (purple), only CDI (red), DAU (yellow), photo-Met (pM, green). Cross-linked K200 of GCAP-2 is not shown as the *C*-terminal region (191-204) is not resolved in the NMR structure. Peptide 2 (cyan, PDB 1AWL) is schematically presented as cartoon structure and placed in the cleft representing a potential interaction site between GCAP-2 and peptide 2.

Chemical cross-linking in combination with MS represents a powerful technique to define interaction sites in protein complexes. In this work, ROS-GC1 peptides 1-3 were cross-linked with GCAP-2 via DSBU, CDI, DAU and photo-Met, reagents which all possess differing reactivities and bridge various distances (from ~8 to 30 Å). The reactivity of specific amino acids is however not only determined by the topology of the protein/peptide complex, but also by local pK_a_ values. In our case, the majority of cross-links were identified between GCAP-2 and peptide 2. Especially for peptide 2, distinct regions were targeted by the different cross-linkers. DSBU cross-links between GCAP-2 and peptide 2 were more frequently observed in the calcium-free than in the calcium-loaded state (Figure 2). As GCAP-2 is more flexible in the calcium-free state, DSBU with a spacer length of 12.5 Å may be able to capture flexible segments, in particular the *C*-terminal part of GCAP-2. In the activating, calcium-free state, CDI mainly connected peptide 2 with the *N*-terminal part of GCAP-2 (G2-K126). Probably, the CDI cross-linker is too short for interactions with the *C*-terminal, flexible region of GCAP-2. CDI exhibited more reactions in the non-activating, tighter, calcium-loaded conformation. However, independent of the calcium-bound state, CDI reacted with four lysines (K96, K98, K102, and K106) in the first α-helix of EF-hand motif 4 (Ames et al., 1999; Figure 5). A single cross-link (C131) with DAU was identified only in the presence of calcium. Residue C131 is located at the beginning of the loop between EF-motifs 3 and 4 and due to a calcium-induced conformational change in this region, cross-linking with DAU seems to be hindered in the absence of calcium. In Figure 5, all residues cross-linked with peptide 2 are highlighted, revealing that several cross-linked amino acids (K46/50, K96/98/102/106, Y125/K126/129, photo-Met181/186, C131) flank a cleft in GCAP-2, which might represent a preferred interaction site for ROS-GC1.

One additional point to be mentioned is the involvement of the peptides' *N*-termini in all cross-linking reactions (Figure 2). This is a drawback of performing cross-linking experiments with short peptides as their *N*-termini do not reflect the true situation in the protein by creating a novel, artificial reaction site for the cross-linker. For peptide 3, only the *N*-terminus was found to be cross-linked to GCAP-2 with DSBU and CDI. Strikingly, both lysine residues in peptide 3, which should preferentially be targeted by amine-reactive cross-linkers, were not cross-linked to GCAP-2 at all. This finding is consistent with SPR and fluorescence measurements and implies a very weak or even missing interaction between GCAP-2 and the ROS-GC region comprising peptide 3.

## Conclusion

In this work, the interaction between GCAP-2 and three ROS-GC1 peptides (Figure 1) was investigated by cross-linking/MS and biophysical analyses. The majority of cross-links were obtained with ROS-GC1 peptide 2 (aa 942-981), in which two segments (residues 1-9, 18-24) reacted preferably. Peptide 2 exhibits the highest affinity for GCAP-2 in the low micromolar range. This result indicates that one GCAP-2 interaction site in ROS-GC1 is located in the region comprising parts of the catalytic domain and the *C*-terminal extension. A few cross-links were identified with peptide 3 (aa 503-522), derived from the juxtamembrane area of ROS-GC1, but no interaction was detected between GCAP-2 and peptide 3 in SPR and fluorescence measurements. Considering conformational transitions of the GCAP-2/ROS-GC1 complex, additional binding regions cannot be ruled out at present, but this issue will be addressed in further investigations using domains as well as full-length ROS-GC1.

## Author contributions

AR, DT, RG, CI, and CHI performed the experiments. AR and AS planned the experiments. AR and AS wrote the manuscript.

### Conflict of interest statement

The authors declare that the research was conducted in the absence of any commercial or financial relationships that could be construed as a potential conflict of interest.

## Abbreviations

CCD: Core catalytic domain
cGMP: Cyclic guanosine monophosphate
CDI, 1: 1′-Carbonyldiimidazole
CMD: Carboxymethyldextran
CTE: *C*-terminal extension
DAU, 1: 3-Diallylurea
DMSO: Dimethyl sulfoxide
DSBU: Disuccinimidyl dibutylurea
DTT: Dithiothreitol
EDC: 1-Ethyl-3-(3-dimethylaminopropyl)carbodiimide hydrochloride
EGTA, Ethylene glycol-bis(2-aminoethylether)-*N, N, N*′: *N*′-tetraacetic acid
ESI: Electrospray ionization
ExtD: Extracellular domain
FA: Formic acid
GCAP: Guanylyl cyclase-activating protein
GTP: Guanosine-5′-triphosphate
HCD: Higher-energy collision-induced dissociation
HEPES: 4-(2-Hydroxyethyl)-1-piperazine ethanesulfonic acid
IAA: Iodacetamide
JmD: Juxtamembrane domain
KHD: Kinase homology domain
LC/MS/MS: Liquid chromatography/tandem mass spectrometry
LS: Leader sequence
MS: Mass spectrometry
NCS: Neuronal calcium sensor
NHS: *N*-Hydroxysuccinimide ester
Photo-Met: Photo-methionine
ROS-GC: Rod outer segment guanylyl cyclase
RP: Reversed phase
SDS-PAGE: Sodium dodecyl sulfate-polyacrylamide gel electrophoresis
SHD: Signal helix domain
SPR: Surface plasmon resonance
TFA: Trifluoroacetic acid
TmD: Transmembrane domain
TRIS: Tris(hydroxymethyl)aminomethane.
