# Supplementary material for: Molecular Details of Retinal Guanylyl Cyclase 1/GCAP-2 Interaction
Source: Front Mol Neurosci. 2018 Sep 19;11:330. doi: 10.3389/fnmol.2018.00330 (PMC6156451; doi:10.3389/fnmol.2018.00330)
Supplement: Supplementary file 1 [file Data_Sheet_1.docx]

Supplementary Material

**Molecular Details of Retinal Guanylyl Cyclase 1/GCAP-2 Interaction**

Anne Rehkamp^1^, Dirk Tänzler^1^, Claudio Iacobucci^1^, Ralph P. Golbik^2^, Christian H. Ihling^1^, Andrea Sinz^1*^

^1^Department of Pharmaceutical Chemistry and Bioanalytics, Institute of Pharmacy, ^2^Section Microbial Biotechnology, Institute of Biochemistry and Biotechnology, Charles Tanford Protein Center, Martin Luther University Halle-Wittenberg, Kurt-Mothes-Str. 3a, Halle (Saale), Germany


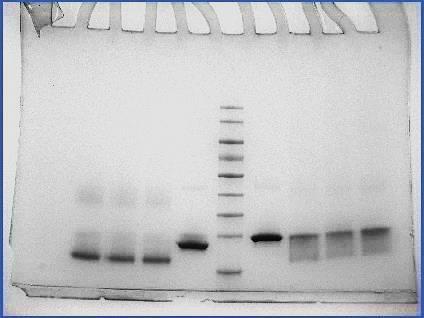


**1 2 3 c**

**c 1 2 3**

**II**

**130 kDa**

**170 kDa**

**15 kDa**

**25 kDa**

**35 kDa**

**55 kDa**

**70 kDa**

**40 kDa**

**100 kDa**

**+ Ca^2+^**

- **Ca^2+^**

**MW**

**Ib**

**Ia**

Supplementary Figure 1 SDS-PAGE of DSBU cross-linking reactions between myristoylated GCAP-2 and ROS-GC 1 peptides 1, 2, and 3 in the presence (+Ca^2+^) and absence of calcium (-Ca^2+^). Lane 1: peptide 1 (aa 965-981), lane 2: peptide 2 (aa 942-981), lane 3: peptide 3 (aa 503-522), and lane c: control sample of mCAP-2 without peptide and cross-linker. Band Ia: GCAP-2 monomer, Ib: GCAP-2/peptide (1:1) complex (for in-gel digestion, bands Ia/b I were concomitantly excised), II: GCAP-2 dimer; MW: molecular weight.

Supplementary Table 1 DSBU cross-linked products between GCAP-2 and the ROS-GC peptides 1-3 in the presence and absence of calcium.

| **DSBU** |  |  |  | |  | |  |  | |  | | |
| --- | --- | --- | --- | --- | --- | --- | --- | --- | --- | --- | --- | --- |
|  |  |  | |  | | **site (1)** | | | **site (2)** | | **calcium** | |
|  | **theor. [M+H]^+^** | **exper.**  **[M+H]^+^** | | **z** | | **ROS-GC1 Peptide** | | | **GCAP-2** | | **with** | **without** |
| **Peptide 1** | 921.552 | 921.551 | | 3 | | *N*-term | | | K128/129 | | ✓ |  |
|  | 983.542 | 983.542 | | 3 | | *N*-term | | | K46 | | ✓ | ✓ |
|  | 983.542 | 983.542 | | 2 | | *N*-term | | | K46 | | ✓ | ✓ |
|  | 1058.611 | 1058.61 | | 3 | | *N*-term | | | K96 | | ✓ | ✓ |
|  | 1058.611 | 1058.61 | | 2 | | *N*-term | | | K96 | | ✓ | ✓ |
|  | 1116.551 | 1116.552 | | 2 | | *N*-term | | | K200 | | ✓ | ✓ |
|  | 1157.61 | 1157.61 | | 3 | | *N*-term | | | K29/30 | | ✓ | ✓ |
|  | 1157.61 | 1157.61 | | 2 | | *N*-term | | | K29/30 | | ✓ |  |
|  | 1272.652 | 1272.652 | | 3 | | *N*-term | | | K200 | | ✓ | ✓ |
|  | 1272.652 | 1272.652 | | 2 | | *N*-term | | | K200 | |  | ✓ |
|  | 1284.731 | 1284.731 | | 3 | | *N*-term | | | K126 | | ✓ | ✓ |
|  | 1284.731 | 1284.731 | | 2 | | *N*-term | | | K126 | | ✓ | ✓ |
|  | 1355.678 | 1355.678 | | 3 | | *N*-term | | | K178 | | ✓ | ✓ |
|  | 1355.678 | 1355.677 | | 2 | | *N*-term | | | K178 | |  | ✓ |
|  | 1454.694 | 1454.695 | | 2 | | *N*-term | | | K129 | | ✓ | ✓ |
|  | 1657.786 | 1657.786 | | 2 | | *N*-term | | | K50 | | ✓ | ✓ |
| **Peptide 2** | 986.582 | 986.579 | | 3 | | *N*-term | | | K128/129 | |  | ✓ |
|  | 986.582 | 986.581 | | 2 | | *N*-term | | | K128/129 | |  | ✓ |
|  | 1048.571 | 1048.57 | | 3 | | *N*-term | | | K46 | |  | ✓ |
|  | 1123.64 | 1123.64 | | 2 | | *N*-term | | | K96 | |  | ✓ |
|  | 1134.598 | 1134.598 | | 2 | | *N*-term | | | K30 | |  | ✓ |
|  | 1139.635 | 1139.635 | | 3 | | *N*-term | | | K96 | |  | ✓ |
|  | 1181.581 | 1181.581 | | 2 | | *N*-term | | | K200 | | ✓ | ✓ |
|  | 1222.64 | 1222.64 | | 2 | | *N*-term | | | K29/30 | |  | ✓ |
|  | 1337.682 | 1337.681 | | 3 | | *N*-term | | | K200 | | ✓ | ✓ |
|  | 1349.761 | 1349.76 | | 3 | | *N*-term | | | K126 | |  | ✓ |
|  | 1349.761 | 1349.762 | | 2 | | *N*-term | | | K126 | |  | ✓ |
|  | 1365.756 | 1365.753 | | 3 | | *N*-term | | | K126 | |  | ✓ |
|  | 1365.756 | 1365.756 | | 2 | | *N*-term | | | K126 | |  | ✓ |
|  | 1404.713 | 1404.713 | | 3 | | *N*-term | | | K178 | |  | ✓ |
|  | 1407.705 | 1407.703 | | 3 | | *N*-term | | | K106 | |  | ✓ |
|  | 1420.708 | 1420.707 | | 3 | | *N*-term | | | K178 | |  | ✓ |
|  | 1423.7 | 1423.699 | | 3 | | *N*-term | | | K106 | |  | ✓ |
|  | 1438.78 | 1438.781 | | 3 | | S19 | | | K46 | | ✓ | ✓ |
|  | 1477.856 | 1477.855 | | 4 | | *N*-term | | | K126 | |  | ✓ |
|  | 1477.856 | 1477.855 | | 3 | | *N*-term | | | K126 | |  | ✓ |
|  | 1519.724 | 1519.722 | | 2 | | *N*-term | | | K129 | |  | ✓ |
|  | 1535.719 | 1535.719 | | 2 | | *N*-term | | | K129 | |  | ✓ |
|  | 1587.786 | 1587.784 | | 2 | | S19 | | | K200 | | ✓ | ✓ |
|  | 1722.816 | 1722.814 | | 2 | | *N*-term | | | K50 | | ✓ | ✓ |
|  | 2410.239 | 2410.241 | | 3 | | *N*-term | | | K142 | |  | ✓ |
|  | 12222.64 | 1222.639 | | 3 | | *N*-term | | | K29/30 | | ✓ | ✓ |

| **DSBU** |  |  |  | |  | |  |  | |  | | |
| --- | --- | --- | --- | --- | --- | --- | --- | --- | --- | --- | --- | --- |
|  |  |  | |  | | **site (1)** | | | **site (2)** | | **calcium** | |
|  | **theor. [M+H]^+^** | **exper.**  **[M+H]^+^** | | **z** | | **ROS-GC1 Peptide** | | | **GCAP-2** | | **with** | **without** |
| **Peptide 3** | 1007.577 | 1007.577 | | 2 | | *N*-term | | | K128/129 | |  | ✓ |
|  | 1069.568 | 1069.568 | | 2 | | *N*-term | | | K46 | | ✓ | ✓ |
|  | 1144.636 | 1144.636 | | 2 | | *N*-term | | | K96 | | ✓ | ✓ |
|  | 1155.593 | 1155.593 | | 2 | | *N*-term | | | K30 | | ✓ | ✓ |
|  | 1202.576 | 1202.576 | | 2 | | *N*-term | | | K200 | |  | ✓ |
|  | 1243.636 | 1243.636 | | 2 | | *N*-term | | | K29/30 | | ✓ | ✓ |
|  | 1358.677 | 1358.678 | | 2 | | *N*-term | | | K200 | | ✓ | ✓ |
|  | 1370.757 | 1370.757 | | 2 | | *N*-term | | | K126 | | ✓ |  |
|  | 1425.708 | 1425.708 | | 2 | | *N*-term | | | K178 | | ✓ |  |
|  | 1540.72 | 1540.721 | | 2 | | *N*-term | | | K129 | |  | ✓ |
|  | 1743.811 | 1743.811 | | 2 | | *N*-term | | | K50 | |  | ✓ |

Supplementary Table 2 CDI cross-linked products between GCAP-2 and the ROS-GC peptides 1-3 in the presence and absence of calcium.

| **CDI** |  |  |  |  |  | | | | |
| --- | --- | --- | --- | --- | --- | --- | --- | --- | --- |
|  |  |  |  |  | **site (1)** | **site (2)** | | **calcium** | |
|  | **theor. [M+H]^+^** | **exper.**  **[M+H]^+^** | **z** | **ROS-GC1 Peptide** | | **GCAP-2** | **with** | | **without** |
| **Peptide 1** | 1487.68 | 1487.68 | 3 | *N*-term | | K50 | ✓ | | ✓ |
|  | 2175.104 | 2175.106 | 3 | *N*-term | | K142 | ✓ | | ✓ |
|  | 2187.984 | 2187.984 | 4 | *N*-term | | K30 | ✓ | |  |
| **Peptide 2** | 1179.655 | 1179.655 | 3 | *N*-term | | K126 | ✓ | |  |
|  | 1179.655 | 1179.655 | 3 | *N*-term | | Y125/K126 |  | | ✓ |
|  | 1234.607 | 1234.608 | 3 | *N*-term | | K178 | ✓ | |  |
|  | 1237.599 | 1237.598 | 3 | *N*-term | | K106 | ✓ | | ✓ |
|  | 1250.671 | 1250.671 | 3 | *N*-term | | K98 | ✓ | | ✓ |
|  | 1401.682 | 1401.683 | 3 | S19/T20/Y24 | | K200 |  | | ✓ |
|  | 1401.683 | 1401.683 | 3 | S19/T20 | | K200 | ✓ | |  |
|  | 1442.743 | 1442.742 | 3 | S19/T20 | | K29 | ✓ | |  |
|  | 1569.864 | 1569.864 | 3 | Y24 | | K126 | ✓ | | ✓ |
|  | 1624.815 | 1624.815 | 4 | Y24 | | K178 | ✓ | |  |
|  | 1640.88 | 1640.879 | 4 | S19/T20 | | K102 | ✓ | | ✓ |
|  | 1640.88 | 1640.881 | 3 | S19/T20 | | K102 | ✓ | |  |
|  | 1739.827 | 1739.831 | 3 | S19/T20 | | K129 | ✓ | |  |
|  | 1799.89 | 1799.888 | 4 | *N*-term | | K102 | ✓ | | ✓ |
|  | 1799.89 | 1799.891 | 3 | *N*-term | | K102 |  | | ✓ |
|  | 1942.918 | 1942.921 | 3 | Y24 | | K50 | ✓ | | ✓ |
|  | 1978.944 | 1978.947 | 3 | Y4 | | K46 | ✓ | |  |
|  | 2054.012 | 2054.013 | 4 | Y4 | | K96 | ✓ | |  |
|  | 2054.012 | 2054.014 | 4 | Y4 | | K96/98 |  | | ✓ |
|  | 2064.969 | 2064.969 | 3 | Y4 | | K30 |  | | ✓ |
|  | 2124.919 | 2124.918 | 3 | *N*-term | | S37 |  | | ✓ |
|  | 2153.012 | 2153.012 | 4 | Y4 | | K29/30 | ✓ | |  |
|  | 2153.012 | 2153.013 | 4 | Y4 | | K29 |  | | ✓ |
|  | 2153.012 | 2153.014 | 3 | Y4 | | K29/30 |  | | ✓ |
|  | 2190.094 | 2190.098 | 4 | S19/T20 | | K102 | ✓ | |  |
|  | 2190.098 | 2190.098 | 4 | Y24 | | K102 |  | | ✓ |
|  | 2240.134 | 2240.134 | 3 | *N*-term | | K142 | ✓ | |  |
|  | 2280.133 | 2280.135 | 4 | Y4 | | K126 | ✓ | | ✓ |
|  | 2338.077 | 2338.079 | 3 | Y4 | | K106 |  | | ✓ |
|  | 2634.236 | 2634.236 | 3 | Y24 | | K50 |  | | ✓ |
| **Peptide 3** | 899.462 | 899.461 | 2 | *N*-term | | K46 |  | | ✓ |
|  | 974.531 | 974.531 | 2 | *N*-term | | K96 |  | | ✓ |
|  | 1271.667 | 1271.668 | 2 | *N*-term/Y2 | | K98 | ✓ | |  |
|  | 1271.667 | 1271.667 | 3 | *N*-term/Y2 | | K98 | ✓ | |  |
|  | 1370.614 | 1370.615 | 2 | *N*-term | | K129 | ✓ | | ✓ |
|  | 1790.936 | 1790.933 | 3 | Y2 | | K106 |  | | ✓ |
|  | 2261.13 | 2261.131 | 3 | Y2 | | K142 |  | | ✓ |

Supplementary Table 3 DAU cross-linked product between GCAP-2 and ROS-GC peptide 2 in the presence and absence of calcium.

| **DAU** | |  |  |  |  | |  | |  | |  |
| --- | --- | --- | --- | --- | --- | --- | --- | --- | --- | --- | --- |
|  |  |  |  | **site (1)** | | **site (2)** | | **calcium** | | | |
|  | **theor. [M+H]^+^** | **exper.**  **[M+H]^+^** | **z** | **ROS-GC1**  **Peptide** | | **GCAP-2** | | **with** | | **without** | |
| **Peptide 2** | 2322.074 | 2322.074 | 3 | C5 | | C131 | | ✓ | |  | |

Supplementary Table 4 Photo-Met cross-linked products between GCAP-2 and ROS-GC peptides 1 and 2 in the presence and absence of calcium.

| **Photo-Met** | |  |  |  |  | |  |  | |
| --- | --- | --- | --- | --- | --- | --- | --- | --- | --- |
|  |  |  |  | **site (1)** | **site (2)** | | **calcium** | | |
|  | **theor. [M+H]^+^** | **exper.**  **[M+H]^+^** | **z** | **ROS-GC1**  **Peptide** | | **GCAP-2** | **with** | | **without** |
| **Peptide 1** | 2996.507 | 2996.507 | 3 | *C*-term | | PM42 |  | | ✓ |
| **Peptide 2** | 1188.656 | 1188.657 | 3 | M1 | | PM181 | ✓ | | ✓ |
|  | 1838.947 | 1838.946 | 4 | E18 | | PM181 | ✓ | |  |
|  | 1390.602 | 1390.604 | 2 | D9 | | PM186 |  | | ✓ |


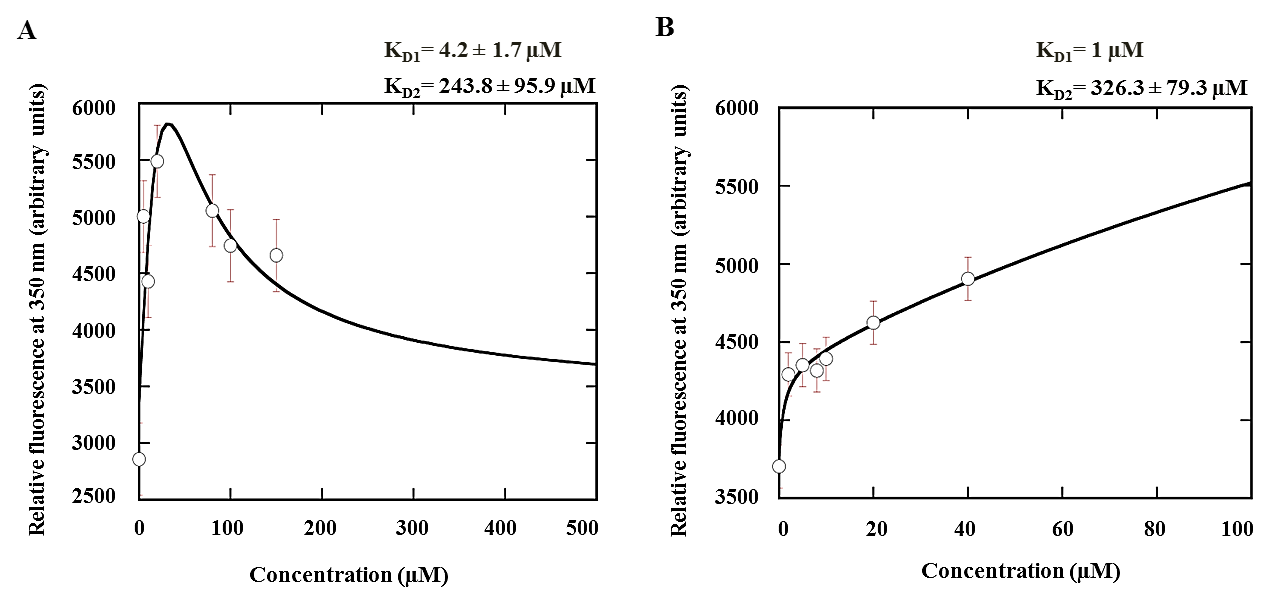


Supplementary Figure 2 Fluorescence spectroscopy measurements to determine the affinities between GCAP-2 and ROS GC-peptides 1 (A) and 2 (B). GCAP-2 was mixed with increasing peptide concentrations; fluorescence emission was determined at 350 nm.

**
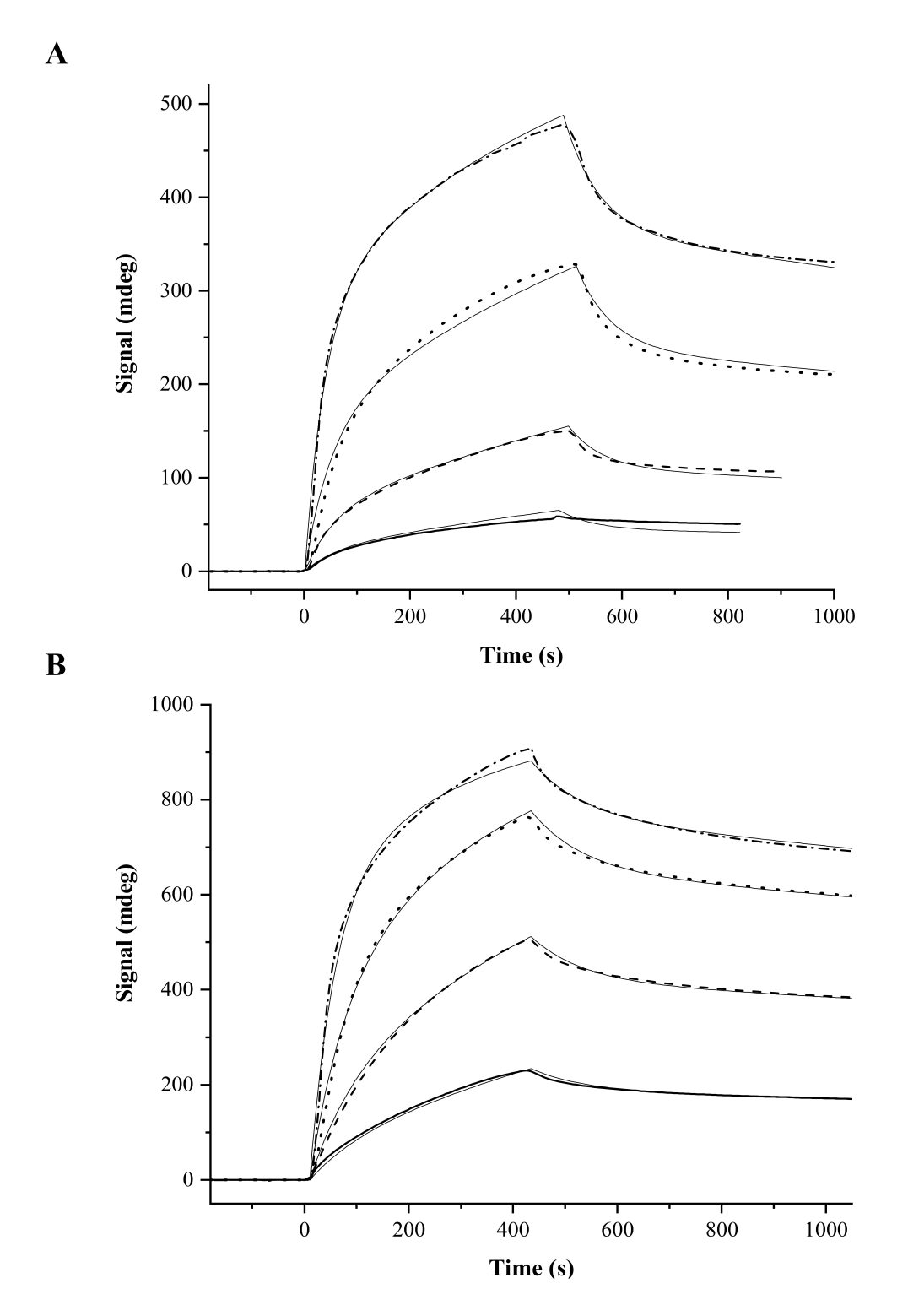
**

**Supplementary Figure 3** SPR measurements between GCAP-2 and ROS-GC **(A)** peptide 1 and **(B)** peptide 2. The following peptide concentrations were applied: 5 µM (solid line), 10 µM (dashed line), 20 µM (dotted line), and 40 µM (dashed/dotted line). Curve fittings are shown as thin, solid lines. The K_D_ value of peptide 1 is 3.6 ± 2.8 µM, while that of peptide 2 is 0.99 ± 0.28 µM.
